# Analysis of hypermethylation and expression profiles of *APC *and *ATM *genes in patients with oral squamous cell carcinoma

**DOI:** 10.1186/1868-7083-3-6

**Published:** 2011-11-01

**Authors:** Mohammad Ayub Rigi-Ladiz, Dor Mohammad Kordi-Tamandani, Adam Torkamanzehi

**Affiliations:** 1 Department of Periodontics, Dental School, Zahedan University of Medical Sciences, Zahedan, Iran; 2 Department of Biology, University of Sistan and Baluchestan, Zahedan, Iran

**Keywords:** *OSCC*, *ATM*, *APC*, DNA methylation, gene expression

## Abstract

**Background:**

*Adenomatous polyposis coli (APC) *and *Ataxia-telangiectasia-mutated *(*ATM*) gene products have an important role in cell cycle control and maintenance of genomic stability. Our aim was to analyze *ATM *and *APC *methylation and its relationship with oral squamous cell carcinoma (OSCC).

**Materials and methods:**

Eighty-four OSCC tissues that have been fixed in paraffin along with 57 control oral samples have been used for analyzing promoter methylation of *ATM *and *APC *genes by Methylation Specific Polymerase Chain Reaction (MS-PCR). In addition, 10 cases of OSCC and the same of matched controls were examined for estimating expression of the above mentioned genes using Real-Time Reverse-Transcription PCR.

**Results:**

Observed promoter methylations were 71.42% and 87.71% for the *APC *gene and 88.09% and 77.19% for the *ATM *gene in cases and controls, respectively. Analysis of these data showed that promoter methylation at *APC *was significantly different in cases compared to healthy controls (*p = 0.01*), but no difference was detected for the *ATM *gene. Furthermore, the mRNA expression levels did not differ statistically between cases and controls for both *ATM *(cases = 9, controls = 10) and *APC *(cases = 11, controls = 10) genes.

**Conclusions:**

Our results, for the first time, provide methylation profiles of *ATM *and *APC *genes in a sample of patients with OSCC in a southeast Iranian population. The present data support related evidence of *APC *methylation effect on OSCC development.

## Introduction

Oral squamous cell carcinoma (OSCC) is one of the most frequently diagnosed types of head and neck cancers based on epidemiological reports [1]. It has been suggested that OSCC can arise in the course of accumulation of multiple genetic alterations [2], for which accurate molecular mechanisms regarding its pathogenesis remain to be understood.

In addition to genetic changes, any alteration in epigenetic information such as DNA methylation, histone modifications, chromatin structure, microRNA, and other genomic functions may make an individual susceptible to developing cancer [3]. Hyper-methylation of promoter CpG islands and repetitive DNA sequences has been recognized as one of the most important variations in the progression of the cancer [4]. Growing data points to the critical role of CpG island hypermethylation in genes implicated in apoptosis, cell cycle regulation, and cellular differentiation in various types of cancer [5,6]. In respect to OSCC development, current studies show that epigenetic silencing of cancer-linked genes through DNA methylation plays a significant role [7,8]. In the genomes of mammals, methylation takes place at CpG islands that are located in the promoter region of approximately half of the genes. These are heritable throughout mitosis and are copied to the new strand by DNA methylatransferase-1 during DNA replication [9]. Although the accurate function of DNA methylation in OSCC is not fully understood, it is well known that gene expression is influenced by DNA methylation. Overall, DNA methylation represses transcription, and loss of methylation restores gene activation [10].

In this study, we examined hypermethylation of *ataxia-telangiectasia-mutated *(ATM) and *adenomatous polyposis coli *(APC) genes and their expression profiles in patients with OSCC.

ATM gene is located on chromosome 11q22-23[11], and encodes a serine-threonine kinase that belongs to the phosphatidylinositol-3 kinase (PI-3K) family. This enzyme plays an essential role in the pathways activated by DNA breaks [12]. In reaction to various agents that damage DNA, ATM phosphorylates p53, which increases its stabilization and expression level, ultimately leading to cell cycle arrest, DNA repair, and apoptosis[13]. However, the possible link between p53 and ATM deactivation in the expansion and development of human tumors is not well established. A number of groups have identified the 11q22-23 locus as a common deletion site in head and neck squamous cell carcinoma (HNSCC)[14]. However, it is presently unclear whether ATM is exhibiting this deletion in the HNSCCs, because this protein has an important role in preserving genomic homeostasis in HNSCC and other types of cancers [15,16].

APC gene is located on chromosome 5q21-q22 and encodes a homodimeric protein that functions in the cytoplasm and nucleus of the cells and has an important role in cell cycle arrest and apoptosis [17]. The wild-type APC protein acts as a vital controller in the Wnt- signaling pathway [18]. A relationship between methylation of CpG sites of *APC *gene and different types of cancer have recently been observed [19,20]. However, the associations of APC and ATM genes' methylation with gene silencing mechanisms have not yet been fully elucidated. The aim of our study was to investigate the effect of hypermethylation of APC and ATM genes and their expression profile in patients with OSCC.

## Results

### Detection of methylation in *APC *and *ATM *genes using MS-PCR

Results of the APC and ATM genes methylation status in patients with OSCC and healthy individuals and their relationship with clincopathalogical parameters are shown in Tables 1 and 2. There was no significant association between methylation status of ATM and APC and clinical parameters such as sex, age, and tumor stages. As shown in table 3, methylation of ATM was found in 88.09% (74 out of 84) of patients with OSCC and in 77.19% (44 out of 57) of healthy controls. Similarly, APC gene methylation was 71.42% (60 out of 84) in cases and 87.71% (50 out of 57) in controls. Comparison of methylation status between cases and controls revealed statistically significant differences for APC (p = 0.01) and insignificant for ATM. This part of result has been analyzed by χ2 and t tests.

**Table 1:** Relationship between *APC *promptor methylation and clinicopathology parameters in healthy controls and cases.

Characteristics	Controls	* *APC *Methylation status		P value	Cases	*APC *methylation status		**P value
	N = 57				N=84			
		Present N (%)	Absent N (%)			Present N (%)	Absent N (%)	
Mean age	39.07 ± 14				54.14 ± 18			
< 50	46	39 (84.8)	7 (15.2)		26	20(76.93)	6 (23.07)	
				0.9				0.2
> 50	11	10(90.90)	1 (9.1)		58	39(67.24)	19(32.76)	

Sex								
Male	21	18 (85.7)	3 (14.3)		39	32(82.05)	7 (17.95)	
				0.06				0.72
Female	36	32(88.88)	4 (11.12)		45	28(62.22)	17(37.78)	

Stage								0.05
I	-	-	-	-	18	11(61.11)	7(38.89)	
II					18	15(83.33)	3(16.64)	
III					43	31(72.08)	12(27.92)	
IV					5	2(40)	3(60)	

* Methylation present: means that PCR product is produced only with methylated primers.

Methylation absent: means that PCR product is produced only with unmethylated primers.

**Chi-square test

**Table 2:** Relationship between *ATM *promptor methylation and clinicopathology parameters in healthy controls and cases.

Characteristics	Controls N = 57	*ATM *methylation status		P value	Cases N = 84	*ATM *methylation status		*P value
						
		Present N (%)	Absent N (%)			Present N (%)	Absent N (%)	
Mean age	39.07 ± 14				54.14 ± 18			
< 50	46	36(78.26)	10(21.74)	0.3	26	23(88.46)	3(11.54)	0.103
> 50	11	8(72.72)	3(27.28)		58	51(87.93)	7(12.07)	

Sex								
Male	21	17(80.95)	4(19.05)	0.6	40	34(85)	6(15)	0.404
Female	36	27(75)	9(25)		44	40(90.90)	4(9.1)	

Stage								
I					18	16(88.88)	2(11.12)	0.465
II	-	-	-	-	18	15(83.32)	3(16.68)	
III					43	38(83.36)	5(11.64)	
IV					5	5(100)	0	

*Chi-square test

**Table 3:** Comparison of promoter methylation of *APC *and *ATAM *genes in patients with oral cavity cancer and healthy controls.

Gene	Methylation statuse	Controls N (%)	Cases N (%)	*P value
*APC*	Present	50 (87.71)	60 (71.42)	0.01
	Absent	7 (12.29)	24 (28.58)	

*ATM*	Present	44 (77.19)	74 (88.09)	0.8
	Absent	13 (22.81)	10 (11.91)	

*Chi-square test

### Analysis of relative *APC *and *ATM *genes expression

Analysis of relative gene expression (2^-ΔΔCT^) for APC and ATM between cases and controls was done by Mann-Witny test. As shown in Table 4, APC relative expression was 2.06 ± 2 for cases (n = 11, range: 0.004-7.4) and 0.95 ± 1.31 for controls (n = 10, range: 0.002-3.43). The ATM data were 0.5 ± 0.9 for cases (n = 9, range: 0.005-2.94) and 1.48 ± 2.2 for controls (n = 10, range: 0.0001-6.27). The difference was not statistically significant between patients and healthy individuals.

**Table 4:** Comparison of gene expression levels (2^-ΔΔCT^) of *ATM *and *APC *between patients with oral cavity cancer and healthy controls.

Gene		N	Mean ± SD	P-value (Mann-Whitney test)
APC	Cases	11	2.06 ± 2.6	0.3
		
	Controls	10	0.95 ± 1.31	
ATM	Cases	9	0.5 ± 0.9	0.9
		
	Controls	10	1.48 ± 2.2	

## Discussion

In this study, our results indicated a significant difference in methylation profile between cases and controls for APC gene (p = 0.01), but not for ATM.

In recent years, gene expression, as well as other molecular profiling, has been used as biological tools for diagnosis of cancer [21,22]. These molecular approaches also have the potential to explore molecular mechanisms of the disease and to pave the road to targeted cancer fighting drugs and advanced treatments [23]. APC is one the most important elements of the Wnt-signaling pathway; its activation is a general characteristic of solid tumors such as bladder, prostate, and renal tumors. Epigenetic down regulation of Wnt pathway inhibitors may contribute to aberrant activation of Wnt signaling pathway [24]. Hypermethylation of APC promoter can silence gene expression by interfering with the binding of transcription factors to the promoter [25]. According, with our results, Uesugi and his colleagues have reported that inactivation of the APC gene via hypermethylation has a significant role in oral carcinogenesis [26].

*Jing et al*., (2010) have also reported an association between hypermethylation of APC gene promoter and breast cancer [27]. Furthermore, there are reports that APC promoter hypermethylation *is *linked with *H. pylori*-associated gastritis [28], cervical cancer [29], and esophageal adenocarcinoma [30]. While our observations support the connection between APC hypermethylation with OSCC progression, this study did not find any significant change in APC expression levels between patients and controls. One explanation for these observation may be due to the involvement of other factors which reducee gene expression. Also, since the number of tumor and control samples analyzed was relatively small it was not enough to establish a strong and significant relationship between hypermethylation and gene expression for these genes. Further analysis involving larger sample sizes are needed to elucidate this matter.

ATM gene is a component of the PI3 kinase family, which is vital in signaling pathways of DNA damage. ATM kinase activates and phosphorylates a number of downstream regulatory proteins that are significant in apoptosis, DNA repair, and cell cycle arrest [31]. Various reports have shown the association of ATM mutation with risk of different human malignancies including breast, prostate, and ovarian cancers, mantle cell lymphoma, and B-cell chronic lymphocytic leukemia. The primary molecular pathway(s) by which ATM gene leads to these alterations are not well known [32]. So far, no study has shown strict involvement of ATM gene methylation with OSCC.

The results of Wai *et al*., (2004) indicated 45% hypermethylation of *ATM *gene in fecal DNA of patients with colorectal cancer and its lack in healthy controls [33]. This has the potential to be used as predictive factor for patients with non-small cell cancer [34] and thyroid carcinoma [35].

Vo QN *et al*., (2004) showed a link between decreased *ATM *function due to epigenetic silencing and sporadic breast malignancy [36]. On the contrary, a study on a full range of B-cell lines with variable degrees of differentiation showed that methylation of *ATM *gene is far less frequent in lymphomagenesis compared to deletions and mutations [37].

DNA methylation and histone modification emerge to work together to silence the expression of a number of genes in cancer, such as APC and hMLH1 [25,38]. However, our study has some limitations, it has been focus on analysis of 2 genes so, identification of further novel CpG islands that are specifically linked with OSCC will be needed to create a panel with higher sensitivity that maintains high specificity, and studies examining detection of such a panel of genes by newly developed quantitative assays should be undertaken.

Obviously, additional studies are needed to further elucidate the relationship between DNA methylation, demethylation, and histone modification in genes regulation. Results from such research may support new strategies and suggest further study with larger sample size for better understanding of molecular mechanisms of cancer, which play a critical role in prevention and management of disease.

## Materials and methods

### Samples and DNA preparation

Eighty-four OSCC tissues that had been fixed in paraffin along with 57 control oral mucosa were collected from patients without history of OSCC who were referred to Periodontics Department, Dental School, Zahedan University of Medical Sciences. All procedures in this study were approved by the Ethical Board at the Zahedan University of Medical Sciences. Genomic DNA was extracted using QIAamp DNA Kits from cancerous (Cat. No. 56404, Qiagen) and healthy control (Cat.No.51304, Qiagen) samples, and then its quality was estimated by a spectrophotometer.

### Methylation-Specific Polymerase Chain Reaction (MS-PCR)

The process of bisulfite conversion of DNA sampleswas conducted as previously described [39]. MSP analysis of promoter regions of *APC *and *ATM *was carried out in 25 μl PCR reactions containing 1 μl of bisulfite-treated genomic DNA, oligonucleotide primers (25 pmol each/reaction), dNTPs (200 μM each), 0.625 units of Hotstar Taq (Qiagen, Valencia, CA) in 1× PCR buffer and 2.5 mM MgCl2. Thermo cycling conditions were carried out using the following settings: 95°C for 10 min; 40 cycles consisting of [30 s at 95°C, 30 s at 61°C for APC (M), 56°C (U) and for ATM 65°C (M), 60°C (U),1 min at 72°C], and a final extension at 72°C for 10 min. Parallel with each set of MSP reactions, positive and negative controls were run. Ten microliters of each MSP reactions were electrophoresed on 3.5% agarose gels and were visualized by ethidium bromide staining. The list of primers were utilized are giving in Table 5.

**Table 5:** Primer sequences and annealing temperature.

Genes	Sequences	Annealing temperature
*APC *M	F:GAACCAAAACGCTCCCCAT R:TTATATGTCGGTTACGTGCGTTTATAT	61

*APC *U	F:AAACCAAAACACTCCCCATTC R:AGTTATATGTTGGTTATGTGTGTTTAT	56

*ATM *M	F:GGAGTTCGAGTCGAAGGG R:CTACCTACTCCCGCTTCCGA	65

*ATM *U	F:GTTTTGGAGTTTGAGTTGAAGGGT R:AACTACCTACTCCCACTTCCAA	60

*GAPDH *(Real Time-PCR)	F: CCACTCCTCCACCTTTGAC R: ACCCTGTTGCTGTAGCCA	60

*APC*(Real Time-PCR)	F:CTTCAAAATTACCTCCAC R:CTCCTCTAACTCCTTCTC	60

*ATM*(Real Time-PCR)	F:CAGGGTAGTTTAGTTGAG GTTGACAG R:CTATACTGGTGGTCAGTGCCAAAGT	60

### Reverse transcription-PCR analysis of *APC *and *ATM*

We extracted total RNA from OSCC and control tissues using the High pure RNA Tissue kit (Cat No: 12033674001) and High pure FFPE RNA Micro kit (Cat No: 04823125001), respectively, according to the manufacturer's instructions. The cDNA synthesis kit (Cat No: K1611, Frmantase) was used for converting 1 μg of total RNA to cDNA according to the manufacturer's instructions. Real time-PCR of APC, ATM, and glyceraldehyde-3-phosphate dehydrogenize (GAPDH) were performed using SYBR green assay by 7300 Real-Time PCR System (Applied Biosystems, Foster City, CA).

### Statistical analysis

Analysis of data was carried out using SPSS software version 17.0 to evaluate association between methylation status of APC and ATM genes and clinical parameters by χ2 and t tests. Assaying relative gene expression (2^-ΔΔCT^) between cases and controls were done by Mann-Whitney test. Significance level was set at P < 0.05 for all tests.

## Competing interests

The authors declare that they have no competing interests.

## Authors' contributions

MR: participated in providing samples, DK: Carried out the molecular genetic studies, the design of the study and the statistical analysis. AT: Participated in its design and coordination. Finally, all authors read and approved the final manuscript.
